# Epigenetic Evidence Implies Disturbed Proteostasis and Potentially Protein Aggregation in Suicidality

**DOI:** 10.3390/biom16050733

**Published:** 2026-05-16

**Authors:** Julija Šmon, Maja Juković, Matea Kršanac, Bobana Samardžija, Alja Videtič Paska, Eva Žerovnik, Katarina Kouter, Nicholas J. Bradshaw

**Affiliations:** 1 Institute of Biochemistry & Molecular Genetics, Faculty of Medicine, University of Ljubljana, 1000 Ljubljana, Slovenia; julija.smon@mf.uni-lj.si (J.Š.); alja.videtic@mf.uni-lj.si (A.V.P.); 2 Faculty of Biotechnology & Drug Development, University of Rijeka, 51000 Rijeka, Croatia; maja.jukovic@biotech.uniri.hr (M.J.); matea.krsanac@biotech.uniri.hr (M.K.); bobana.samardzija@biotech.uniri.hr (B.S.); 3 Department of Biochemistry and Molecular and Structural Biology, Jožef Stefan Institute, 1000 Ljubljana, Slovenia; eva.zerovnik@ijs.si; 4 Jožef Stefan’s International Postgraduate School, 1000 Ljubljana, Slovenia; 5 Institute of Microbiology & Immunology, Faculty of Medicine, University of Ljubljana, 1000 Ljubljana, Slovenia

**Keywords:** suicide, proteostasis, protein aggregation, epigenetics

## Abstract

Suicide is a major public health concern and cause of death worldwide. While progress has been made in understanding molecular pathways involved in suicide, much more work is needed to identify clinically useful biomarkers of suicidality. Disturbed cellular proteostasis and aggregation of specific misfolded proteins are established pathological factors of neurodegenerative diseases. Increasing evidence also suggests that such aggregates often occur in patients with chronic mental illnesses. Recently, genes related to disturbed proteostasis showed differential methylation in individuals who died by suicide compared to controls. These include five genes encoding proteins that aggregate in neurodegenerative and/or mental illness: *CRMP1* (also called *DPYSL1*), *DISC1*, *MAPT* (encoding the Tau protein), *PRKN* (also called *PARK2*, encoding Parkin), and *SOD1*. Given the possibility that altered methylation in these genes could affect expression of the proteins they encode, we aimed to review evidence for whether disturbed proteostasis may be a point of overlap between suicidality, neurodegenerative disease, and/or mental illnesses. Epigenetic changes in most of these genes also occur in other neurological disorders. Autophagy, and, to a lesser extent, the ubiquitin–proteasome system, are emerging as potentially impaired in individuals with suicidal tendencies and individuals who died by suicide. Based on this accumulated data, we hypothesise that disturbed proteostasis is likely to be a pathological component of suicidality. It is also plausible that this may lead to the accumulation of aggregated proteins in a similar manner to, and potentially overlapping with, those seen in major mental illnesses. If true, this would have consequences for potential identification of biomarkers for suicidality and should be a priority for future research in the field.

## 1. Introduction—The Pathophysiology of Suicide

Suicide is a major public health concern, accounting for more than 700,000 deaths globally every year. Suicide attempts are even more frequent, as it is estimated that suicide attempts are up to 20 times more frequent than completed suicides [1]. Suicide refers to death caused by a voluntary and intentional self-directed act [2], while suicidality, or suicidal behaviour, also includes suicide attempts [3]. Approximately 50% of violent deaths in men and 70% in women are caused by suicide [4], making it the fourth leading cause of death among 15–29 year olds [1]. In over 90% of cases, suicide is associated with an underlying mental illness. Notably, patients with major depressive disorder (MDD) have up to 20 times greater risk of suicide than the general population [2]. Suicidality is a personal tragedy and also deeply affects families and communities [1]. Efforts have been made toward its prevention; however, they have been met with insufficient success [5]. Suicidality is known to be influenced by biological, psychological, clinical, social, and environmental risk factors [3,6]. Our knowledge of these factors is still insufficient to have predictive value; therefore, novel biomarkers for risk of suicide are greatly needed [7].

As suicidality is a complex, etiologically heterogeneous disorder, the success of preventative measures relies on collaborative effort among multiple societal sectors [1,8]. This proves challenging for various reasons, including the poor availability and quality of data, stigma relating to mental illnesses [1], and our incomplete understanding of the neurobiology of suicidality [2]. Animal models of human suicidality are very limited [9] and, due to the difficulties of in vivo approaches in humans, many studies rely on post-mortem samples that cannot provide longitudinal data [2]. Multi-omics approaches may facilitate biomarker discovery; however, these require complex computational tools [10].

Many of these issues are also shared by research of major mental illnesses. It is known, however, that in at least a subset of patients with MDD, schizophrenia (SCZ), and bipolar disorder (BPD), diagnosis is associated with the presence of specific protein aggregates in the brain [11,12,13]. These aggregates are similar to those seen in neurodegenerative diseases, although the proteins involved are mostly distinct between the two classes of disease, and notably, the protein aggregates implicated in mental illness do not have the same level of neurotoxicity as those in neurodegenerative disease.

Recently, we looked at methylation in the hippocampi of individuals who died by suicide, relative to control individuals [14]. Upon revisiting the data, it became clear that, among the genes showing altered methylation in individuals who died by suicide, were several that encoded proteins prone to aggregation in major mental illness and/or neurodegenerative disease (see Section 4.1), with some of them also showing altered transcript expression [15]. While not yet proven, alterations in transcript expression could lead to altered expression of protein in these brain regions. This leads to the intriguing concept that proteostasis of these proteins could be altered in the brains of individuals with suicidality. While protein aggregation has not yet been specifically studied in suicidal behaviour, there is evidence of changes in systems such as the ubiquitin–proteasome system and autophagy. Such evidence of disturbed proteostasis in people with suicidal tendencies would be consistent with the potential presence of protein aggregation in those individuals. Inspired by these results and other recent research on the biological basis of suicide, particularly including its common correlation with MDD, we put forward a two-part hypothesis: firstly, that disturbed proteostasis is an aspect of the pathology of suicidal behaviour, as has been seen in MDD, and secondly, that epigenetics is involved in this.

## 2. Current State of Biological Research into Suicidality

To estimate suicide risk, various models of suicidality have been developed, considering proximal (triggering), distal (predisposing), and developmental risk factors [3]. According to the stress-diathesis model, suicide occurs as a result of the interaction between environmental stressors and a susceptibility or diathesis that is independent of mental illness [16]. Epidemiologic studies of families, twins, and adoptees have estimated that approximately 40% of the risk of suicidal behaviour may be attributed to biological factors, which was further supported by genome-wide association studies [2]. While a large number of genes have so far been associated with an increased suicide risk, their estimated individual contribution is low [2]. Attempts to elucidate the neurobiological correlates of suicide diathesis have strongly implicated roles of the serotonergic system, the hypothalamic–pituitary–adrenal (HPA) axis, and, to a lesser degree, the noradrenergic and dopaminergic systems, focusing mostly on brain areas involved in decision-making, emotional regulation, and memory [17]. Recently, glutamatergic system dysregulation has also been postulated as a therapeutic target [18], and the role of (neuro)inflammation as a trigger for various downstream effectors, predisposing to suicide diathesis, has been proposed [19,20]. Similar advances have been made in the field of neuroimaging studies. Structural and functional alterations in the brain have been linked to suicidal behaviour, correlating to neurochemical deficits in regions involved in emotion and impulse regulation [21]. The identification of distinct biological suicide subtypes has been at the forefront of suicidality research, with the aim of improving the accuracy and efficiency of diagnostic and therapeutic procedures [22].

### 2.1. Epigenetic Studies

Epigenetic modulation presents a link between environmental influences, such as childhood adversity, and genetic predisposition [23]. Animal models have shown that maternal neglect and chronic stress may promote excessive stress response in adulthood through methylation of the glucocorticoid receptor gene promoter region, which suppresses its transcription [24,25]. In individuals who died by suicide and also reported childhood trauma, similar findings were observed, as well as decreased glucocorticoid receptor gene (*NR3C1*, nuclear receptor subfamily 3, group C, member 1) expression in the hippocampus [26]. In the context of suicidality, the majority of human epigenetic studies have focused on DNA methylation in post-mortem brain samples; less frequently, DNA methylation has been studied in leukocytes [27]. A meta-analysis has shown alterations in DNA methylation patterns in the prefrontal cortex and cerebellum of individuals who died by suicide compared to controls; the differentially methylated genetic loci corresponded to pathways involved in nervous system development and synapse function [28]. In a recent epigenome-wide association study, differential methylation patterns in *SLC4A2* (solute carrier family 4 member 2), *CDK5* (cyclin-dependent kinase 5), *PDE3A* (phosphodiesterase 3A), and *RARRES3* (retinoic acid receptor responder 3) were associated with suicidal behaviour in veterans [29].

DNA methylation represents only one layer of cytosine modification. For example, DNA hydroxymethylation, particularly 5-hydroxymethylcytosine, is abundant in the brain and may also contribute to neuronal gene regulation. Compared with DNA methylation, DNA hydroxymethylation is less well studied and is present at high levels in Purkinje cells. This is significant because 5-methylcytosine (5mC) and 5-hydroxymethylcytosine (5hmC) may have different associations with transcription, and some commonly used DNA methylation methods may not fully distinguish between 5mC and 5hmC. Therefore, when studies report altered DNA methylation in brain disorders, these findings should be interpreted as evidence of altered epigenetic regulation, not as evidence that 5mC alone is responsible for further changes in gene expression or proteostasis [30].

Micro-RNAs (miRNAs), which play an important role in neurodevelopment, have also been implicated in suicide; however, findings have been inconsistent [31,32]. Fewer studies have examined histone modification patterns in individuals who died by suicide [23]. While epigenomic research can provide insight into suicidality-associated changes in the regulation of gene expression, their downstream effects can be examined via other techniques, including proteomic analyses.

### 2.2. Proteomics Studies

Proteomic profiling of post-mortem brain tissue from individuals who died by suicide and individuals with mood disorders supports the involvement of protein dysregulation in suicidal behaviour. Hundreds of proteins were seen to be differentially expressed in the prefrontal cortex of individuals who died by suicide, compared to controls, including prominent downregulation of pathways related to apoptotic signalling and protein localization [33]. Key proteins such as CAPNS1 (calpain small subunit 1), CSNK2B (casein kinase 2β), and PTP4A2 (protein tyrosine phosphatase 4A2), linked to neuroprotection, apoptosis, and neuroinflammation via the NF-κB (nuclear factor κB) pathway, were implicated. Another study found alterations in the dorsolateral prefrontal cortex proteome of individuals who died by suicide with mood disorders, notably involving the GABAergic signalling pathway, which is crucial for inhibitory neurotransmission and mood regulation [34].

Monsalve et al. (2014) reported altered expression patterns of Notch receptors and their downstream effectors in the dorsolateral prefrontal cortex and amygdala of individuals who died by suicide, suggesting that disruptions in neuronal development and plasticity contribute to the pathophysiology of suicide [35]. These findings implicate the Notch signalling pathway, a key regulator of neural cell proliferation, differentiation, and synaptic plasticity, in the maladaptive changes observed in the brains of individuals who died by suicide, particularly in regions critical for emotion regulation and cognitive control.

Several studies have investigated the role of brain-derived neurotrophic factor (BDNF), nerve growth factor (NGF), and their receptors in the neurobiology of suicide, particularly focusing on post-mortem brain tissue [36]. BDNF, a member of the neurotrophin family, is crucial for the development, survival, and plasticity of neurons in the central nervous system. It acts through its receptor TrkB (tyrosine kinase receptor B), while NGF signals via TrkA (tyrosine kinase receptor A). Research consistently shows that individuals who died by suicide exhibit significantly reduced mRNA and protein expression levels of BDNF, NGF, and their cognate receptors in key brain regions such as the hippocampus and prefrontal cortex compared to non-psychiatric controls [37,38]. Decreased concentrations of BDNF and NGF proteins were also seen in hippocampal tissue from suicide patients, alongside reduced expression of their receptors TrkB and TrkA, detected via Western blot. These reductions were mirrored at the mRNA level, suggesting a transcriptional downregulation associated with suicide [37]. Lower BDNF and TrkB mRNA and protein levels were seen in another study in both the prefrontal cortex (Brodmann area 9) and hippocampus of 27 suicide subjects compared to 21 controls, reinforcing the notion of impaired neurotrophic support in suicide [39]. A systematic review and meta-analysis by Eisen et al. [40] further corroborated these findings, highlighting that decreased BDNF levels in post-mortem brain regions such as the prefrontal cortex and hippocampus are consistently observed in individuals who died by suicide relative to controls. However, some brain areas, like the entorhinal cortex and amygdala, did not show significant differences, indicating region-specific alterations.

The converging evidence from protein and gene expression studies in post-mortem brains indicates that diminished neurotrophic support via BDNF, NGF, and their receptors plays a significant role in the pathophysiology of suicide. These findings implicate impaired neuronal survival, synaptic plasticity, and neurogenesis in suicide and suggest potential molecular targets for therapeutic intervention aimed at restoring neurotrophic function. More generally, however, the fact that several proteins are differentially regulated in individuals who died by suicide opens the possibility that protein regulation, and therefore proteostasis, may also be affected in suicidality. In both neurodegenerative diseases and major mental illnesses, disturbances in the maintenance of proteostasis are known in the brains of patients, leading to the formation of protein aggregates [11,12,13].

## 3. Protein Aggregation

### 3.1. Types of Protein Aggregation

**Figure 1:**
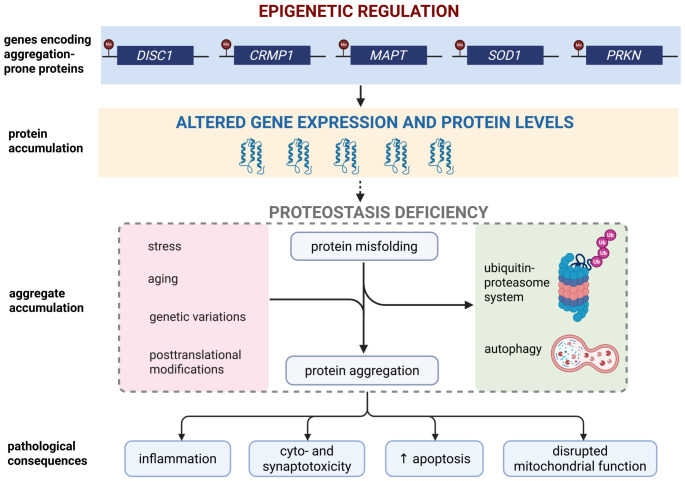
Mechanisms contributing to disturbed proteostasis in brain disorders. Genetic variations can increase risk for protein aggregation, as is exemplified by familial cases of neurodegenerative diseases. Epigenetic regulation (e.g., DNA methylation, schematically represented by methyl groups attached to gene promoters) influences gene transcription and, consequently, has the potential to ultimately affect protein levels. Stress, aging, and various posttranslational modifications may promote protein misfolding and aggregate formation. If physiological cellular defence mechanisms, such as autophagy and the ubiquitin–proteasome system, are overwhelmed or deficient, then these can lead to protein aggregates accumulating. Such aggregates have the potential to exert harmful influences on neuronal function. Recent research has suggested that the accumulation of specific protein aggregates in the brain is not limited to neurodegenerative disorders but can also occur in mental disorders and may be relevant for suicidality research. Created in BioRender. Šmon, J. (2026) https://biorender.com/e06afpt (accessed on 19 March 2026). Abbreviations: CRMP1, collapsin response mediator protein 1. DISC1, disrupted in schizophrenia 1. MAPT, microtubule-associated protein tau. Me, methyl group. SOD1, superoxide dismutase 1. PRKN, parkin. Ub, ubiquitin.

Protein aggregation refers to the misfolding of proteins, leading them to accumulate in large multimeric structures in or outside of the cell. This process normally takes place when cells encounter stress, and is counteracted by various cellular defence systems, including the ubiquitin–proteasome system and autophagy. Typically, such processes are sufficient to degrade any misfolded protein and maintain correct proteostasis. When these systems are overwhelmed by high levels of aggregation, however, or if they are impaired in some manner, then excess misfolded protein can accumulate into aggregates. There are at least two kinds of protein aggregation pathways: the deposition pathway, leading to amyloid fibrils or unfolded aggregates, and the condensation pathway, leading to protein condensates [41,42]. These interrelated processes are summarized in Figure 1, which illustrates the multifactorial mechanisms that disrupt proteostasis in brain disorders. It was discovered that under appropriate conditions, any protein can form amyloid fibrils [43], with intrinsically disordered proteins being more prone than folded proteins to form amyloid structures. Pre-fibrillar aggregates are believed to be cytotoxic, acting similarly to protein toxins, that is, they can bind/perforate membranes [44,45] composed of acidic phospholipids, cholesterol, and gangliosides [46]. Amyloids also have functional roles, including the ability to store melatonin and hormones, as well as help in building long-term memory [47,48].

Following the condensation pathway, intrinsically disordered proteins in particular condense into various droplet states: granules and inclusion bodies [49], also called membrane-less organelles, which are important for cellular regulation [50,51]. The condensed bodies can be found in the cytoplasm and the nucleus, where different molecules, including proteins, RNAs, and small molecules, co-exist [50,52]. Protein aggregation, although considered to be irreversible and harmful, may act as a beneficial cellular response under extreme conditions such as nutrient scarcity, elevated temperatures, and oxygen deprivation. In these cases, protein aggregates communicate the stress encountered by the cell and, by various defence mechanisms such as helper proteins (chaperones) and degradation systems, together restore proteostasis [53,54]. The propensity to aggregate may also be increased by post-translational modifications or environmental factors, including chemicals or ultraviolet light. This can also lead to damaged autophagic flux, damaged lysosomal/endosomal pathway, and finally to damaged mitochondria with increased oxidative stress.

Aging plays a significant role as a risk factor for protein misfolding diseases, including neurodegenerative ones. With aging, a decreased level of energy and decreased efficiency of protein folding quality control mechanisms contribute to increased protein aggregation and less efficient clearance of misfolded protein. These changes have therapeutic implications. For example, if the quality control machinery could be protected from the effects of aging, it may be possible to significantly delay the onset of protein misfolding diseases. On the other hand, acute stress can be helpful in delaying aging. Heat shock response and unfolded protein response start a cascade of events leading to cell renewal and actually may contribute to longevity [55].

### 3.2. Protein Aggregation in Neurodegenerative Disease

Protein aggregation is a hallmark of neurodegenerative diseases, including Alzheimer’s disease (AD), Parkinson’s disease (PD), and amyotrophic lateral sclerosis (ALS), and understanding the biological and physicochemical mechanisms behind such aggregation could lead to improved therapies [56,57]. The causal relationship between protein aggregates and downstream consequences in these conditions is difficult to prove. Familial cases with mutated proteins, however, which are more aggregate-prone, seem to imply that protein aggregation is, or can be, an initial causal event. For any protein that aggregates, one must look at two distinct sets of consequences: those caused by loss of function of the normally folded protein and those caused by gain of toxic function of the aggregated protein.

In AD there are two major aggregating proteins: Tau, a microtubule-associated protein, and the amyloid-β peptide (Aβ), which is 40 or 42 amino-acids long and derived by proteolysis from the amyloid precursor protein (APP, amyloid precursor protein). Functionally, Aβ may play a crucial role in synaptic plasticity (long-term potentiation) that underlies learning and memory [58]. Other physiological functions suggested for Aβ include acting as a chelating agent, promoting recovery from injuries, antimicrobial activity, and decreasing oxidative stress [57]. Amyloid plaques, made from Aβ fibrils and accompanying proteins, are extracellular and may be protective because they sequester the more toxic forms of this peptide. Explanations for Aβ toxicity have developed from the original amyloid cascade hypothesis [59]. It is hypothesised that soluble, oligomeric species, which interact with and even perforate cellular membranes, may act as synaptotoxins [60]. Tau, encoded by the *MAPT* (microtubule-associated protein Tau) gene, exists in six isoforms (ranging from 352 to 441 amino acid residues), resulting from alternative splicing [61]. Functionally, Tau is a stabilizing microtubule-associated protein [62]. When hyperphosphorylated, it forms neurofibrillary tangles, which are composed of filaments forming a β-structured core. In contrast to Aβ, aggregation of Tau has been directly implicated to decreased cognitive function [63].

*PRKN* (parkin RBR E3 ubiquitin protein ligase, also called *PARK2*) encodes parkin, a protein of 465 amino acid residues. Parkin functions as an E3 ubiquitin-protein ligase, attaching ubiquitin moieties to proteins, thus marking them for degradation by the ubiquitin–proteasome system. It also ubiquitinates surface proteins on damaged mitochondria, marking them for mitophagy, that is, degradation of mitochondria by autophagy. Mutations in the *PRKN* gene cause a familial form of PD known as autosomal recessive juvenile PD. Parkin interacts with many other proteins, for example, with α-synuclein, a key aggregating protein in PD. Furthermore, a PDZ domain (PSD95, Dlg1, and zo-1 domain) containing scaffolding protein CASK/Lin2 (calcium/calmodulin-dependent serine protein kinase/liprin-2) interacts with the PDZ binding motif of parkin. The PDZ domain is a common structural domain, containing 80–90 amino acids. Proteins that contain PDZ domains play an important role in the process of anchoring receptor proteins in the membrane to cytoskeletal components [64]. A network of PDZ-interacting proteins has the potential to form a complex web of molecules that surround parkin and regulate its function. PINK1 (PTEN-induced kinase 1) is a serine/threonine kinase that activates parkin by phosphorylating it. PINK1 mutations also cause autosomal recessive juvenile PD by disabling mitophagy [65].

ALS is a fatal neurodegenerative disease affecting motor neurons. It appears in sporadic and familial form. In familial cases of ALS, the proteins SOD1 (superoxide dismutase 1), C9ORF72 (chromosome 9 open reading frame 72), TDP-43 (TAR DNA-binding protein 43), and FUS (fused in sarcoma) have each been seen variously to aggregate [66]. Human SOD1 is a 32 kDa homodimeric protein localized in the cytosol, nucleus, and intermembrane space of mitochondria [67]. SOD1 mainly acts as an antioxidant by scavenging reactive oxygen species, which catalyses the reaction of superoxide radicals to hydrogen peroxide and molecular oxygen [68]. Recently, it has also been shown that SOD1 may possess additional roles in modulating metabolism and regulating transcription [69]. Mutations of SOD1 causing familial ALS impair mitochondrial function, with consequences for mitochondrial fission and fusion, as well as mitophagy clearance [68]. Protein aggregation has therefore been thoroughly characterized in neurodegenerative disease. More recently, however, the role of protein aggregation in another group of chronic brain disorders, mental illnesses, is coming to be appreciated.

### 3.3. Protein Aggregation of DISC1 and CRMP1 in Mental Illness

Multiple studies have indicated that proteostasis is disturbed in the brains of patients with SCZ. Notably, a general increase in protein insolubility was identified among a subgroup of SCZ patients [70], which can also be detected in olfactory cells grown from living patients [71]. While protein insolubility can arise from multiple factors, when a normally soluble protein becomes insoluble, it can indicate that it has formed an aggregate. In support of this, cellular mechanisms that would normally destroy such aggregating proteins are seemingly less functional, on average, in SCZ patients, with brain samples of patients showing reduced proteasomal activity [72] and increases in the total level of ubiquitinated protein relative to controls [70,73]. Proteomic analyses of synapses from patients with SCZ and BPD also show a reduction in proteins linked to autophagy [74]. Unusually large levels of protein aggregation and/or a reduced ability to degrade the formation of such species therefore appear to be an aspect of SCZ pathology.

Aggregation of specific proteins has also been observed in patients with mental illness, and these proteins are distinct from the proteins associated with neurodegenerative disease [11]. This could be because these proteins are particularly vulnerable to aggregation, meaning that the general deficits in proteostasis are prone to causing these specific proteins to aggregate. It could also be that aggregation of these specific proteins, for example, due to mutation, is precipitous of the general overloading of the proteostasis machinery. These aggregating proteins include CRMP1 (collapsin response mediator protein 1), DISC1 (disrupted in schizophrenia 1), dysbindin-1 (dystrobrevin-binding protein 1, encoded by *DTNBP1*), and TRIOBP (trio and F-actin binding protein), each of which has been variously detected in patients with SCZ, BPD, and/or MDD [75,76,77,78,79]. Additionally, NPAS3 (neuronal PAS domain protein 3), FABP3, and FABP7 (fatty acid binding proteins 3 and 7) are also implicated through rare aggregation-inducing mutations in patients [80,81,82].

The *DISC1* gene is a well-known risk factor for major mental illness, originally identified from a single family, many members of which possessed a t(1;11) chromosomal translocation that caused loss of one functional copy of *DISC1*. This translocation was strongly linked with MDD, SCZ, and other mental illnesses [83,84]. Functionally, DISC1 is a ubiquitously expressed scaffold protein that interacts with, and modulates, a wide variety of proteins that are of importance for neurodevelopment and synaptic functioning, particularly in dopaminergic systems [85,86,87,88,89].

The DISC1 protein was found to be insoluble in a subset of post-mortem brain samples (Brodmann area 23, posterior cingulate cortex) from patients with SCZ, MDD, and BPD, but not in matched controls, implying aggregation [75]. Since this initial test, DISC1 aggregation has been seen in a wide variety of experimental systems [11], including recently in the cerebrospinal fluid of SCZ patients [90]. The most detailed analysis of the effect of DISC1 aggregation comes from a transgenic rat model, which expresses human DISC1 across the brain, leading to the formation of DISC1 aggregates that can be detected both by immunofluorescence microscopy and through insoluble protein purification [91]. These animals show a range of behavioural changes, of which the most robust are altered reactions to amphetamine [91,92,93] and decreased sociability [94,95]. Changes in neuroanatomy [96] and neuroelectrophysiology [97] are also reported. In many of these experiments, dopaminergic pathways are directly implicated [91]. Curiously, the altered sociability can also be seen in *DISC1* transgenic fruit flies [98]. These findings indicate that DISC1 aggregation may be a causal event in mental illness, as opposed to an effect of illness, should these results be translatable to humans. Cell culture and other in vitro model systems have shown that DISC1 may aggregate via multiple mechanisms, with two distinct sections of the protein, each capable of forming aggregates [99,100,101]. DISC1 can also be induced to aggregate in cells by the addition of dopamine [91]. The mechanistic consequences of DISC1 aggregates at a cellular level are largely unexplored, but include an ability to disrupt the movement of mitochondria [102]. DISC1 aggregates were also seen to be cell-invasive in multiple systems, suggesting the ability to pass from cell to cell in the brain, in a phenomenon resembling the spreading and infectivity of prions and amyloid proteins [76,103].

In contrast to *DISC1*, the *CRMP1* gene was not previously considered a risk factor for any major mental illness. It was instead identified as potentially aggregating in mental illness through an unbiased proteomics approach, which identified it as existing in an insoluble state in one or more patients with SCZ, but not in a matched set of controls [77]. It was then confirmed to be found in an insoluble state in subpopulations of brain samples of patients with SCZ, BPD, and MDD [77]. The CRMP proteins are cytosolic phosphoproteins, whose expression is generally limited to the nervous system, where they participate in pathways involving semaphorins, short-range inhibitory signalling molecules, crucial as axonal growth cone guidance molecules [104]. Additionally, CRMP1 is involved in Reelin signalling mediation and regulation of neuronal migration, as proven by *Crmp1* −/− mice. In Crmp1-deficient mice, there are reduced levels of neuronal migration in the cerebral cortex [77], as well as a decrease in long-term potentiation, spatial learning, and memory [105]. All this indicates that CRMP1 could play an important role in the process of brain development and synaptic plasticity.

It was noticed that aggregates of DISC1 and CRMP1 proteins often co-exist in the brains of the same patients [77,106]. In cell culture, CRMP1 is seen to interact directly with DISC1, and is “pulled into” its aggregates, in a process of co-aggregation [77,106]. This potential interaction of DISC1 and CRMP1 in mental illness is also seen at the genetic level, with alleles of *CRMP1* being associated with specific endophenotypes of SCZ, but only on the background of a specific *DISC1* genotype [77]. Curiously, both DISC1 and CRMP1 co-aggregate with huntingtin (encoded by *HTT*), the protein that forms aggregates in Huntington’s disease [107,108]. DISC1 has also been found to co-aggregate with the protein TDP-43 in frontotemporal lobe dementia [109], together showing partial overlap in the pathologies of mental illness and neurodegenerative disease.

The mechanisms by which aggregation of DISC1 and CRMP1 occurs in the brain are currently unknown. By analogy to proteins in neurodegenerative diseases, it is likely to be a combination of cellular stress and/or rare mutations. Recent progress in identifying aggregated proteins in more accessible tissues than the brain [71,110,111], including the specific identification of DISC1 in the cerebrospinal fluid of patients [90], suggests potential translational value to the clinic for detecting biomarkers of certain patient subpopulations. Furthermore, strategies aimed at disaggregating pathogenic proteins, such as those targeting TDP-43 or tau in ALS and AD, may be applied in the context of psychiatric disorders.

It is interesting to note that while DISC1 and CRMP1 have been implicated as aggregating in multiple mental illnesses, neither shows specificity to a single diagnosis. Instead, both have been seen to aggregate in brain samples from patients with SCZ, BPD, and MDD [75,77,106]. This implies that aggregation of DISC1 and CRMP1 may be related (either as a causative element or as a symptom) to fundamental processes underlying mental illnesses generally, at least in a subset of patients. Given the frequent comorbidity and at least partially shared pathology of suicide with mental illness, it is therefore very interesting to consider the possibility that DISC1, CRMP1, or similar proteins, may also show protein aggregation in suicidality.

## 4. Epigenetic Overlap Between Suicide and Genes Involved in Protein Aggregation

### 4.1. Altered Methylation of Genes Encoding Proteins Known to Form Aggregates, in Individuals Who Died by Suicide

Suicidality is a complex phenotype, but it also commonly overlaps with mental illness. Based on a meta-analysis of 27 studies on suicide, it was reported that over 80% of subjects had a diagnosis of a mental illness before death [112]. However, this is an extreme case, as in the literature we can also find studies showing much smaller percentages of psychiatric diagnoses among subjects who died by suicide, ranging from 30–80%. The most prevalent diagnosis is mood disorder, followed by schizophrenia, anxiety disorder, and substance abuse [113]. The association between neurodegenerative disorders and suicide is relatively rarely studied, but an extensive cohort study is available through a Danish register. Among all suicide deaths from 1980 to 2016, it was determined that the suicide rate was significantly higher among subjects with a diagnosis of a neurological disorder (such as dementia, amyotrophic lateral sclerosis, or Huntington disease) in comparison to subjects without such a diagnosis [114]. A large population-based cohort study is also available for PD, and again, the risk for suicide was significantly higher among patients with PD compared to the control group [115]. However, neurodegenerative disorders alone are not an important risk factor for suicide but become one at the time of diagnosis and in earlier stages of the disease. Furthermore, it is important to bear in mind that comorbidity of neurodegenerative disorders with mood disorders and anxiety is an additional risk factor and often the link to suicidality.

If disturbed proteostasis, or protein aggregation more specifically, does link the pathogeneses of mental illness and suicidality, it should be investigated whether disruption in upstream regulatory mechanisms contributes to increased aggregation propensity of individual proteins. Such aggregation could arise as a result of alterations in protein expression or in mechanisms involved in protein turnover. In either case, a potential effect of misregulation would be the accumulation of excess protein in the cell, which brings with it the risk of protein aggregation. In this section, we focus on the latter of these situations, and specifically on the role of epigenetics in suicidal behaviour, which has the potential to lead to alterations in protein expression changes, while the more general proteostasis maintenance systems will be discussed in Section 5.

Multiple studies have evaluated DNA methylation in association with suicidal behaviour, including one done by some of the authors of this review [14]. In that study, we looked at genome-wide DNA methylation status in the hippocampus and Brodmann area 9 of individuals who died by suicide, as well as control group subjects. Comparing differentially methylated cytosine bases, numerous sites were identified as differentially methylated. Gene ontology analysis revealed an association with semaphorin signalling, cell structural integrity, and nervous system regulation. As is common in such experiments, significant amounts of data, in this case, lists of differentially methylated genes, were produced. We therefore revisited our existing data with specific attention to genes that could possibly be associated with protein aggregation. Notably, altered methylation was seen in multiple genes, including *DISC1*, *CRMP1*, *MAPT, PRKN* (also known as *PARK2*, encoding Parkin), and *SOD1*, all of which encode proteins known to aggregate in major mental illness and/or neurodegenerative disorders (more details provided in Table 1). As general gene ontology was done at the time of analysis and, as no specific attention was given to protein aggregation at that time, these genes were initially overlooked [14,15]. As an initial follow-up to this, expression of many of these genes was examined in two brain regions of individuals who died by suicide, compared with controls. In the hippocampus, those who died by suicide showed significantly higher expression of *CRMP1*, *SOD1,* and *PRKN*, while significantly reduced levels of *MAPT*, whereas in Brodmann area 46, they showed significantly lower levels of *DISC1* [15].

It is important to highlight that the Reduced Representation Bisulfite Sequencing method, which was used in the study of Kouter et al. [14], is based on a conventional bisulfite treatment, which, after conversion of unmethylated cytosines to uracil, cannot distinguish between DNA methylation and hydroxymethylation [116]. This is a common limitation of the interpretation of DNA methylation studies, as most available studies, including many genome-wide DNA methylation analyses, do not specifically address DNA hydroxymethylation. Therefore, differential signals at cytosine sites within or near *DISC1, CRMP1, MAPT, PRKN*, and *SOD1* should be interpreted with caution: they may reflect altered 5mC, altered 5hmC, or a combination of both, depending on the method used. Although direct evidence linking (hydroxy)methylation of these genes to protein aggregation in suicide is currently lacking, future studies should distinguish 5mC from 5hmC when investigating the epigenetic regulation of aggregation-prone proteins and proteostasis-related pathways.

**Table 1:** Protein aggregation-associated genes that were differentially methylated in individuals who died by suicide compared to control group subjects in a study by Kouter et al. [14].

Gene	Location of CpGs	Status in Individuals Who Died by Suicide [14]	Protein	Associated with Disturbed Proteostasis in
*DISC1*	Gene intron	Hypomethylation of four CpGs	DISC1	Schizophrenia, bipolar disorder, major depressive disorder [75], Huntington’s disease [108], frontotemporal lobe dementia [109]
*CRMP1* (*DPYSL1*)	Gene intron Gene promoter 1 to 5 kb upstream of the TSS	Hypomethylation of six CpGs Hypermethylation of two CpGs	CRMP1	Schizophrenia, bipolar disorder, major depressive disorder [76], Huntington’s disease [107]
*MAPT*	Gene intron Gene exon	Hypomethylation of six CpGs	Tau	Alzheimer’s disease, Pick’s disease, progressive supranuclear palsy, corticobasal degeneration, and others [117]
*PRKN* (*PARK2*)	Gene intron	Hypomethylation of one CpG	Parkin	Juvenile Parkinson’s disease [118]
*SOD1*	Gene promoter	Hypomethylation of two CpGs	SOD1	Amyotrophic lateral sclerosis [119]

This opens the intriguing possibility that proteostasis of the proteins encoded by these genes may also be disrupted in individuals with suicidal tendency, or even that they, too, form aggregates in this condition, as they do in other brain disorders. Given that many proteins can be seen to aggregate in multiple mental illnesses and/or neurodegenerative diseases, it is plausible to predict the relevance of disturbed proteostasis to suicidality as well. For the remainder of this review, we will therefore evaluate evidence that may link suicidality to disturbed proteostasis and the consequences of this for research into suicidality.

Given that we are hypothesising changes in epigenetic modification of these genes to correlate with disturbed proteostasis in suicide, it is reasonable to conclude that epigenetic regulation of these genes would also be disturbed in the diseases in which their proteins are known to aggregate. As a first step, we therefore look at whether similar epigenetic evidence also associates these genes with mental illnesses and/or neurodegenerative diseases. This is also summarized in Table 1.

### 4.2. Epigenetic Regulation of DISC1

Khan et al. investigated DNA methylation of the *DISC1* promoter region in a mouse model of SCZ [120]. Pregnant mice were treated with methyl-azoxy-methanol (MAM), which is known to cause SCZ-like symptoms in offspring, without directly affecting the methylation of cytosines. Gene expression and methylation status were then compared in the cingulum and prefrontal cortex of pre-adolescent and post-adolescent MAM mice, with an additional control group of mice (mothers treated with vehicle agent only). Increased DNA methylation of *DISC1* was observed in pre-adolescent mice, compared with both post-adolescent and control mice. The lowest gene expression, however, was observed in post-adolescent MAM mice, with no clear correlation between DNA methylation and gene expression. Given the active role of DISC1 in neuroplasticity, neuronal growth, and development, the authors hypothesise that increasing *DISC1* DNA methylation could serve as a way of protecting against or slowing down inappropriate synapse and neuronal development [120].

Merging four different Gene Expression Omnibus (GEO) datasets, Zou et al. tried to construct a DNA methylation model of SCZ. The datasets contained information on DNA methylation in various brain regions in SCZ patients and healthy controls. One of the top 10 common differentially methylated genes involved in neuropsychiatric processes was *DISC1* [121].

*DISC1* DNA methylation was also assessed in the blood of the original t(1;11) translocation family, in which the *DISC1* gene was first identified, looking at members either with (N = 17) or without (N = 24) the t(1;11) translocation that causes loss of a functional *DISC1* gene. All carriers of the translocation had a confirmed mental disorder diagnosis, while in the noncarrier group, 18 were unaffected. Out of 13 differentially methylated sites, four of them were located in the *DISC1* and were hypomethylated in family members with the translocation [122].

### 4.3. Epigenetic Regulation of CRMP1

While there are proteomic studies indicating that the CRMP1 protein aggregates in mental disorders, to our knowledge, no study has so far examined DNA methylation of the *CRMP1* gene in regard to psychiatric states. The status of DNA methylation of *CRMP1* has been investigated, however, in association with cancer, where increased DNA methylation levels of a *CRMP1* promoter were observed in positive lymph node breast cancer tissues [123]. Similarly, increased DNA methylation has been observed in hepatocellular carcinoma patients [124]. This could indicate less protein, but no additional studies regarding gene or protein expression were made.

### 4.4. Epigenetic Regulation of MAPT

The Tau protein, known for its involvement in neurodegeneration, is encoded by the *MAPT* gene, located on chromosome 17. Genetic variations in this gene can contribute to the onset of Tau-related dementia [125], and a growing number of epigenetic studies also suggest a link to DNA methylation.

Chromosome 17 carries a large, roughly 900 kb inversion polymorphism 17q21.31, and this genomic region also includes the *MAPT* gene. 17q21.31 is present in two distinct haplotypes, H1 or H2. Haplotype H1 has been associated with progressive supranuclear palsy and frontotemporal dementia. When Li et al. investigated DNA methylation in the blood of patients with neurodegeneration compared to healthy controls, they observed changes in DNA methylation levels [126], which notably clustered within the 17q21.31 region. Next, they compared carriers of the H1 and H2 haplotype, regardless of the health state of the subject, and observed that H1 carriers had significant changes in the DNA methylation status of *MAPT.* The mechanism by which H1 increases the neurodegeneration risk could therefore be partly due to epigenetic regulation [126]. DNA methylation of *MAPT* has also been investigated in relation to altered sleep patterns and mental health state in children. DNA methylation patterns in the 17q21.31 region, including *MAPT*, have been associated with sleep duration, but no association was observed with mental states of the paediatric subjects [127].

Less common are studies that focus on DNA methylation in samples other than blood. Iwata et al. analysed DNA methylation in the post-mortem brain of AD subjects compared to a control group. Pyrosequencing revealed changes in *MAPT* DNA methylation in sporadic AD subjects, with notable alterations of DNA methylation both in neuronal and non-neuronal cells, leading to increased gene expression of *MAPT* [128]. *MAPT* was also identified as a gene of interest in an in-silico study, examining the publicly available data set of DNA methylation and gene expression of patients with age-related macular degeneration. Bioinformatic tools were then used to identify genes having both significant gene expression and DNA methylation data in blood and eye tissue samples of these patients. *MAPT* was identified as such a gene, and when further building a protein-protein interaction model, MAPT was one of the top hits [129].

Finally, *MAPT* DNA methylation appeared to be altered in sperm samples of men who experienced childhood abuse (physical, emotional, or sexual). A differentially methylated region within the *MAPT* gene body showed increased methylation in the group that suffered abuse, compared to the control group. This suggests a possible epigenetic link between early life trauma and the regulation of genes such as *MAPT*, possibly linking trauma and neurodevelopment to neurodegeneration [130]. This is important as multiple studies on animal models [24,131], as well as in humans, have associated early-life adversity to altered DNA methylation of various genes, most notably those involved in the HPA axis [25].

### 4.5. Epigenetic Regulation of PRKN

To the best of our knowledge, no studies have investigated DNA methylation levels of *PRKN* in psychiatric conditions. DNA methylation of the *PRKN* gene has been studied in neurodegeneration, especially regarding PD, but the studies often presented inconclusive results. In a small pilot study, Navarro-Sanchez et al. investigated DNA methylation in three brain regions of 5 PD patients and 5 controls. One of the targeted genes was also *PRKN*. There was a significant alteration in DNA methylation of specific CpGs located in the *PRKN* promoter region, but such results were only observed in one of the studied brain regions (substantia nigra) and had a low percentage difference between PD and the control group [132]. Similar results of decreased DNA methylation, but in blood, were observed in early-onset PD, where *PRKN* promoter DNA methylation levels were investigated on a larger sample size (91 early-onset PD patients compared to 52 healthy controls) [133].

### 4.6. Epigenetic Regulation of SOD1

SOD enzymes play a key role in the antioxidant system, since they convert superoxide into molecular oxygen or hydrogen peroxide, and also reduce levels of nitrous oxide (NO). NO is an important intra- and intercellular messenger and also has neuroprotective functions at physiological levels. However, NO can cause lipid peroxidation through the reaction with superoxide and formation of peroxynitrite. Therefore, the levels of SOD and NO have to be maintained at physiological concentrations [134,135]. Oxidative stress and lipid peroxidation have been shown to be changed in depression, and also in completed and attempted suicide. In young individuals who died by suicide, oxidative stress markers, as well as the size and number of cells in the pituitary gland, which is part of the HPA stress axis, were determined [134]. Compared to controls, increased weight of the anterior-pituitary region was found in individuals who died by suicide, which has been previously shown in subjects with MDD [136], and also reduced levels of NO and higher activity of SOD [134]. In MDD and SCZ patients with suicidal tendencies, a significant decrease in SOD activity in the blood was determined [137]. Similarly, decreased levels of *SOD* gene expression were present in the central nervous system of individuals who died by suicide with MDD compared to controls [138]. In SCZ patients, excess oxidative stress and changes in antioxidant enzymes have been repeatedly shown. Studies of SOD activity have been inconsistent, showing both reduction and increase; however, recent meta-analysis showed stable SOD activity in blood [139].

Epigenetic regulation of *SOD* has been shown indirectly. Namely, in late-onset AD patients’ blood, hyper-methylation of the promoter region of the gene encoding repressor element 1 silencing transcription factor (REST) was present in comparison to healthy controls, thus leading to lower levels of REST. At the same time, diminished levels of the antioxidant enzyme SOD, which could be regulated by REST, were present in the same patients. REST appears to be an intriguing target, as it serves as a key transcriptional repressor of neuron-specific genes in non-neuronal cells and neuronal progenitors [140]. DNA methylation of SOD was investigated in ALS patients. In comparison to their healthy family members, patients exhibited hypomethylation [141]; however, the gene expression data or SOD activity were not available in this study to further support the SOD function in these patients.

In light of this accumulating evidence of altered epigenetic regulation of genes encoding aggregation-prone proteins in brain disorders, our next step is to examine disrupted proteostasis in relation to psychopathologies and, specifically, suicidality.

## 5. Disrupted Proteostasis and a Possible Role of Proteinopathy in Suicide

### 5.1. Hypothesis: The Potential for Disturbed Proteostasis, Potentially Including Protein Aggregation, in the Pathophysiology of Suicidality

The results of our studies [14,15] and these other epigenetic analyses (discussed in Section 4) allude to changes in DNA methylation within genes capable of protein aggregation, both in suicidality and other brain disorders. One possible consequence of these changes in epigenetic regulation of these genes could be altered expression of the proteins they encode. While such protein expression changes are not yet proven, these findings suggest a possible link between suicidality, epigenetic regulatory mechanisms, and disturbances in proteostasis. Epigenetic mechanisms, including the best-studied of them, DNA methylation (and potentially others, such as DNA hydroxymethylation), can significantly influence gene expression itself and thus the levels of proteins produced. These changes do not provide direct evidence of protein aggregation. Instead, they point to possible upstream regulatory changes that could affect how vulnerable cells are to proteostatic stress. Altered regulation of aggregation-prone proteins, especially in the presence of cellular stress, impaired autophagy, ubiquitin–proteasome dysfunction, oxidative stress, or age-related decline in protein quality control, may favour protein misfolding and accumulation. For example, altered epigenetic regulation of *MAPT* or *DISC1* may affect tau or DISC1 expression, but whether this contributes to protein aggregation in suicidality remains to be tested experimentally.

We therefore hypothesise that suicidality can also be considered a potential proteinopathy, that is, a condition defined by the presence of one or more proteins that form aggregates in the brain. Specifically, we predict that disrupted proteostasis of specific proteins, likely caused through a combination of genetic, epigenetic, and environmental factors, can cause specific proteins to form insoluble protein aggregates in the brain. Protein misfolding and subsequent aggregation are central hallmarks of neurodegenerative diseases, but in recent years, there has been a growing recognition of their involvement in psychiatric disorders such as schizophrenia and MDD. Genetic epilepsies also show increased protein aggregation [142]. As protein aggregation seems to span a range of diagnoses, it is therefore plausible to predict its relevance to suicidality as well. The specific proteins that aggregate in suicidality are not yet determined; however, given that many of the proteins that aggregate in major mental illness cross traditional diagnostic boundaries [11], it is plausible that at least some of them may also aggregate in suicidality.

In addition to epigenetic overlap, two other lines of evidence back up this suggestion: evidence linking suicide to the protein clearance pathways, and cognitive decline in co-morbidities of suicidality.

### 5.2. Supporting Evidence for Disturbed Proteostasis in Suicide

So far, only a few studies have looked at potential protein aggregation in the brains of people who died by suicide, and these were done in order to answer larger questions on aggregation of these proteins, not as a systematic examination of those who died by suicide compared to controls [106,143]. While several individuals displayed moderate levels of protein insolubility, two individuals were identified with high levels (at least 20-fold higher than average) of insoluble DISC1 or TRIOBP-1, respectively, in their brains [143]. If disruption of proteostasis, seen in individuals with suicidal tendency, is a more general phenomenon, however, then we would expect to see such individuals with abnormal activity of the main systems involved in degrading misfolded proteins: the ubiquitin–proteasome system and/or the autophagy–lysosome pathway. Impairment of the autophagy–lysosome pathway, alongside the ubiquitin–proteasome system, contributes to neurodegeneration in diseases like PD [144]. Similar mechanisms of autophagy–lysosome pathway dysfunction appear relevant to mental illnesses, where disrupted clearance leads to toxic protein accumulation and cellular stress. Macroautophagy involves the formation of double-membraned autophagosomes that fuse with lysosomes for degradation, and its disruption leads to accumulation of autophagic vacuoles and lysosomal dysfunction observed in some neuropsychiatric conditions [145,146,147].

It is important to note that, ideally, such disturbed proteostasis would be detected in the brain itself, but for practical reasons, it is more common to look in peripheral tissues such as the blood. If the disturbed proteostasis results from genetics and/or external stress factors, then it is reasonable to hypothesis that anomalies in the proteostasis of one tissue would likely be accompanied by equivalent changes in other tissues.

Several studies have documented increased apoptosis in patients with MDD, particularly those who are also at a high risk for suicide. Eilat et al. provided preliminary evidence of elevated apoptosis in MDD patients, which was later supported by Szuster-Ciesielska et al. [148,149], who found accelerated apoptosis of blood leukocytes and increased oxidative stress in these individuals. Amidfar et al. extended these findings by demonstrating that peripheral blood mononuclear cells from those MDD patients specifically with high suicide risk exhibit even greater apoptotic activity than MDD patients generally [150]. These apoptotic changes are linked to altered expression of key regulatory proteins, including Bcl-2 (B-cell lymphoma), Bax (Bcl-2-associated X protein), and FADD (FAS-associated death domain protein) [145,146,147], suggesting that dysregulated cell death pathways may compromise neuronal survival and synaptic integrity in MDD and suicide.

Work from several research groups has shown increased apoptosis in peripheral blood mononuclear cells of MDD patients with a high suicide risk. Oxidative stress connects to the Nrf2-NMDA (nuclear factor erythroid 2-related factor 2-N-methyl-D-aspartate) receptor pathway in the brains of individuals who died by suicide, suggesting that oxidative stress responses and their alterations in this pathway may play a role in suicidal behaviour [151]. Furthermore, converging evidence suggests that serotonergic dysfunction may mediate the relationship between alcohol consumption and AD. Excess alcohol consumption is suggested to accelerate AD development via direct effects on serotonergic function. AD is associated with extensive serotonergic deficits in numerous brain regions, which might partly explain the agitation and irritability associated with the disease [152]. Studies have also indicated the involvement of the serotonergic system in learning and memory, suggesting it may modulate the effects of alcohol on higher mental functions. Serotonergic neurons and neurotransmitter loss in normal aging and neuropsychiatric diseases of late life may contribute to behavioural changes commonly observed in the elderly population [153].

Building on this, Szuster-Ciesielska et al. investigated specific leukocyte subpopulations and apoptotic mechanisms in 29 MDD patients and 30 controls [149]. They found accelerated apoptosis predominantly in CD4+ T cells and CD14+ monocytes, with these cells showing increased expression of the death receptor Fas and the pro-apoptotic protein Bax. Although anti-apoptotic Bcl-2 levels were comparable to controls, the Bcl-2/Bax ratio was significantly reduced in CD14+ cells, indicating a shift toward apoptosis. Moreover, increased release of cytochrome c, a key mitochondrial apoptotic factor, was detected in the serum of depressed patients, particularly from polymorphonuclear cells, underscoring mitochondrial involvement in apoptosis in MDD. Amidfar et al. [150] extended these findings by examining mRNA expression of apoptotic regulators Bcl-2, Bax, and Fas in peripheral blood mononuclear cells of MDD patients stratified by suicide risk. They reported significantly reduced Bcl-2 and increased Fas mRNA expression in MDD patients compared to controls, with the most pronounced changes observed in patients at high risk for suicide. Bax mRNA was significantly elevated only in the high-risk group. Consequently, the Bcl-2/Bax ratio was markedly decreased in MDD patients, especially those with heightened suicide risk. These results implicate both intrinsic (mitochondrial) and extrinsic (death receptor-mediated) apoptotic pathways in MDD, with greater apoptotic dysregulation correlating with suicide risk.

Evidence for disturbances in the ubiquitin–proteasome system in suicide is scarcer. A large-scale genome-wide association study for suicidal thoughts, however, conducted in US military veterans by Kimbrel et al., showed significant enrichment for polymorphisms in genetic loci related to ubiquitination processes [154].

### 5.3. Supporting Evidence Due to Cognitive Decline in Comorbidities

Several studies report that individuals with comorbid conditions, specifically MDD and substance use disorders, are at higher risk for suicide ideation and attempts compared to individuals diagnosed with only one of these conditions. This supports the idea of there being underlying partially shared pathologies between these conditions and suicidality. Particularly in instances where the mental health condition is triggered or associated with an outside stress factor or trauma, there is the possibility that this is associated with a disbalance in proteostasis.

An American study assessing comorbidities between mental disorders and suicidal behaviour, performed by Nock et al. [155], reported that approximately 80% of individuals who attempt suicide have a prior diagnosable mental disorder. In their study, mood disorders, particularly MDD and BPD, showed the highest odds ratios for suicide attempts, followed closely by anxiety disorders, impulse-control disorders, and substance use disorders. Furthermore, they demonstrated that, while depression is a strong predictor of suicide ideation, progression to suicide plans and attempts is more closely associated with the anxiety-inducing disorders, post-traumatic stress disorder, and/or poor impulse control. Similar findings were discussed in other national studies elsewhere, such as in Brazil and South Korea [156,157].

Interestingly, substance abuse was also found to be one of the strongest predictors of suicide attempts [158]. Toxicological analyses revealed widespread involvement of both prescription and illicit drugs in suicide deaths. Trends in substance use vary geographically, with pesticide poisoning being prevalent in low- and middle-income countries. In contrast, most detected substances in suicide deaths in the high-income Western countries include psychotropic, benzodiazepines, alcohol, and opioids [159,160]. Co-occurrence of substance abuse disorders and psychiatric disorders elevates the risk of suicide, even more prominently in some cohorts such as veterans [161]. Another behavioural comorbidity record in suicide is gambling disorder, which further confirms the hypothesis that impulsivity and addictive behaviour may elevate suicide risk [162,163]. Smoking is also associated with higher suicide rates compared to the suicidal tendencies of non-smoking individuals [158].

Other contributing factors include socioeconomic and environmental determinants, such as unemployment rate, social isolation, and food insecurity. The latter is linked to increased suicidal ideation and tendencies indiscriminately among age groups and independent of other socioeconomic factors [164,165,166]. Therefore, suicide risk is multifactorial and significantly elevated in present comorbidities, such as psychiatric disorders, substance abuse, and adverse socio-environmental conditions.

### 5.4. Potential Consequences of Proteostasis Disruption for Treatment in Suicidality

Our hypothesis claims that proteostasis may be disrupted in suicidal individuals, in a similar way to subsets of patients with major mental illness, as a result of multiple mechanisms, which may include the changes in epigenetic regulation of genes encoding aggregation proteins. This has been seen so far in a small number of individuals [143], but not yet investigated systematically. Notably, there is a considerable overlap between individuals with a diagnosis of mental illness and those who die by suicide, and a similar overlap exists in the underlying biology of both of these groups. The presence of suicidality can therefore be considered a means of defining a subset within major mental illness patients. There is therefore great value in pursuing specific biomarkers of suicidality within patients with MDD and similar mental illnesses, and in determining to what extent disturbed proteostasis overlaps with these endophenotypes.

**Figure 2:**
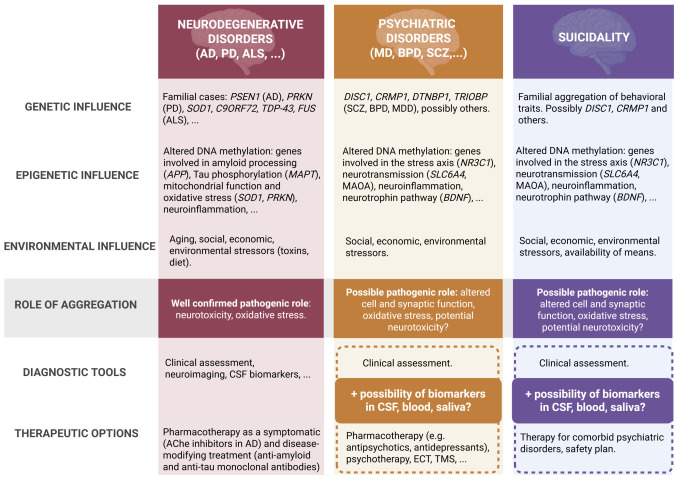
Multi-level comparison of brain conditions with confirmed or hypothesised disturbance of proteostasis. Most brain disorders are complex, with unclear aetiology and limited treatment options. Multiple factors, including genetic, epigenetic, and environmental, contribute to their development (Firdaus and Li, 2024, [168]; Turecki, 2014, [169]). Out of the three disease categories presented in columns, protein aggregation and its role in disease pathogenesis is most researched in neurodegenerative disorders (e.g., AD, PD, ALS). Rare but severe cases of neurodegenerative disorders have been linked to familial clustering of pathogenic variations (Bertram and Tanzi, 2005, [170]; Raguseo et al., 2023, [171]). In psychiatric disorders, however, proteins that aggregate are distinct from those of neurodegenerative disorders. Many mental illnesses and suicidality show a strong hereditary component, and while familial clustering is present (e.g., DISC1 disruption by a balanced translocation, t(1;11) (q42. 1;q14. 3), co-segregates with SCZ, BPD, and MDD), causative genes have been harder to identify (Millar et al., 2000, [83]). Recent research has suggested the involvement of protein aggregation in suicidality, possibly driven by stress and epigenetic modifications. Further investigation of the scope, causes, and consequences of this phenomenon may provide novel tools (e.g., biomarkers) for risk stratification and targeted psychiatric therapy. Created in BioRender. Šmon, J. (2026) https://biorender.com/yqpfsnk (accessed on 19 March 2026). Abbreviations: AChE, acetylcholinesterase. AD, Alzheimer’s disease. ALS, amyotrophic lateral sclerosis. APP, amyloid precursor protein. BDNF, brain-derived neurotrophic factor. BPD, bipolar disorder. CRMP1, collapsin response mediator protein 1. CSF, cerebrospinal fluid. C9ORF72, chromosome 9 open reading frame 72. DISC1, disrupted in schizophrenia 1. DTNBP1, dystrobrevin binding protein 1 (dysbindin). ECT, electroconvulsive therapy. FUS, fused in sarcoma. MAOA, monoamine oxidase A. MAPT, microtubule-associated protein tau. MDD, major depressive disorder. NR3C1, nuclear receptor subfamily 3 group C member 1. PD, Parkinson’s disease. PRKN, parkin. PSEN1, presenilin 1. SCZ, schizophrenia. SLC6A4, solute carrier family 6 member 4. SOD1, superoxide dismutase 1. TDP-43, TAR DNA-binding protein 43. TMS, transcranial magnetic stimulation. TRIOBP, TRIO, and F-actin binding protein.

Protein aggregation increases with aging and leads to proteotoxicity. Age is also a risk factor for vascular dementia and AD, as well as PD, all of which are often accompanied by depression. Other even more fatal neurodegenerative diseases, such as AD and Huntington’s disease, are often accompanied by psychotic states. As most of these illnesses are multi-factorial, not only gene mutations but also sporadic and environmental changes can represent a trigger. It can be assumed that chronic (physiological or psychological) stress to neurons may contribute to protein aggregation. The persistent stress present in suicidality may well translate into disbalance in neuronal proteostasis. Figure 2 shows a comparison of the disruption of proteostasis in neurodegenerative diseases, psychiatric disorders, and suicidality and illustrates both the established and emerging role of protein aggregation in brain pathology. Numerous biomarkers of protein aggregation have been suggested for neurodegenerative disease (Table 2 and references therein). If a biological link between disrupted proteostasis and suicidality can be established, as predicted by our hypothesis, then such biomarkers could potentially be studied also in relation to suicide. Some studies have begun to do this. Mamdani et al. identified potential blood biomarkers associated with suicide in individuals with MDD, reinforcing the notion that molecular alterations in peripheral tissues reflect central nervous system pathology [167]. However, looking for protein aggregates and other biomarkers connected to disrupted proteostasis, albeit appearing temporarily, would be important to follow in the blood and saliva of people with MDD and/or suicidality. Collectively, these findings from genetic, molecular, and clinical studies illustrate that suicide risk arises from complex interactions among genetic predisposition, neurobiological dysfunction, and behavioural traits such as impulsivity and risk-taking. The identification of specific genetic variants and apoptotic mechanisms offers promising avenues for developing biomarkers of disrupted proteostasis in the brain. If, as we hypothesise, disturbed proteostasis and/or protein aggregation affect suicidality, then biomarkers of this sort may ultimately have value in the fields of early detection and targeted interventions for suicide prevention in MDD, or potentially more generally.

Indeed, some compounds already exist that act in relation to disturbed proteostasis and may therefore have future utility for mental illness and/or suicidality. For example, metformin, which is primarily used to treat type 2 diabetes, was recently introduced to delay cognitive decline, dementia, and even stroke [172]. It can have other beneficial effects also in MDD and SCZ, to fight weight gain caused by antidepressant and antipsychotic drugs. Another such drug is rapamycin and its derivatives, which influence mTOR (mechanistic target of rapamycin)-dependent autophagy. Together with mTOR-independent autophagy inducers, it ameliorates toxicity in proteinopathies. It also decreases protein aggregates, reduces neuroinflammation, and prevents demyelination [173]. One could also follow the usage of drugs acting on neuroinflammation, such as ACE (angiotensin-converting enzyme) inhibitors and various antidepressants, which are known to be stress-suppressing. Some new possible drugs still in ongoing trials are: peroxisome proliferator-activated receptor (PPAR) agonists, which inhibit NF-κB activation in the PPAR-dependent pathway. The agonists of PPAR were shown to prevent post-stress neuroinflammation and oxidative/NO damage, including inducible NO synthesis inhibition, NF-κB blocking, TNF-α (tumour necrosis factor α) emission inhibition, and reduction in COX-2 (cyclooxygenase-2) expression [174]. Inhibition of COX-2 has been shown to improve neuroinflammation and depression [175]. NMDA receptor antagonist reduces oxidative and NO damage by inhibiting excessive glutamate in cells and also improves neuroinflammation. While the potential utility of these compounds in suicidality has not been studied, they make an exciting line for future research.

## 6. Conclusions

In recent years, increasing evidence has arisen that mental illnesses, to some extent, overlap in pathology with neurodegenerative disease. This is particularly apparent in the emerging recognition that disturbances in proteostasis are common, particularly in SCZ, and with aggregation of specific proteins having been detected in a variety of mental illnesses. While suicidality is a distinct clinical condition to mental illnesses such as SCZ and MDD, there is significant overlap in their pathology, as can be seen in proteomic, genetic, and epigenetic approaches. Our recent findings show altered methylation and expression of genes encoding aggregation-prone proteins in the brains of individuals who died by suicide compared to controls. Such epigenetic regulation is likely, but not yet demonstrated, to affect expression of the proteins they encode. We therefore put forward a two-part hypothesis. Firstly, based on multiple lines of evidence, including comorbidity findings and evidence of altered apoptosis in cells, including neurons, of individuals who died by suicide and individuals at high risk of suicide, we hypothesise that disturbed proteostasis, with or without protein aggregation, plays a role in the biology of suicidal behaviour. This likely overlaps with the emerging role for similar changes in proteostasis in the biology of MDD and other mental illnesses that are comorbid with suicidality. Secondly, we hypothesise that the changes in methylation and transcript expression of genes encoding known aggregation proteins may represent an upstream step in the development of such pathological changes in proteostasis. It should be emphasised, that the role of proteostasis dysfunction, potentially including protein aggregation, remains to be proven experimentally, as alterations in methylation patterns of genes may not necessarily lead to changes in expression of the proteins they encode.

A key limitation of the current body of evidence is the largely indirect link between epigenetic alterations and disturbed proteostasis in suicidality. DNA methylation changes are not inherently indicative of corresponding changes in protein expression or downstream effects on proteostasis. Furthermore, the observed epigenetic alterations are not necessarily specific to suicidality. They are, however, also present across a range of neuropsychiatric disorders. Therefore, it is possible that they reflect shared stress- or disease-associated processes rather than mechanisms unique to suicidality. As an alternative interpretation, these epigenetic signatures may represent secondary effects of chronic stress, inflammation, medication exposure, or comorbid psychiatric disorders, and may not function as drivers of proteostasis disruption. Finally, as most of the available data are derived from post-mortem or peripheral tissues, insight into temporal dynamics is limited. These considerations highlight the need for integrative, longitudinal, and multi-omics studies to clarify whether epigenetic alterations contribute directly to impaired proteostasis in suicidality. Future work aiming to identify biomarkers of suicidal tendency should therefore also investigate proteostasis. Such hypothetical future biomarkers could look at general indications of disturbed proteostasis, such as changes in proteasomal or autophagic activity, or levels of protein ubiquitination, as well as investigating the possibility that specific proteins, such as CRMP1 and DISC1, may form aggregates in individuals with suicidal tendency, in a similar manner to subgroups of individuals with major mental illness. In terms of clinical consequences, this theory suggests that some medications associated with neurodegenerative disorders, such as AD, could also be tested as complementary or add-on therapies for suicidal tendency. In the longer term, identification of proteostasis and/or protein aggregation biomarkers may have utility for the identification of suicide risk and personalised therapeutic approaches towards treating it.

## Figures and Tables

**Table 2:** Potential biomarkers associated with disturbances in proteostasis, based on studies of other diseases. Should the link between suicidality and protein aggregation be experimentally established, then approaches such as these could also be useful in the investigation of suicidal tendency.

Biomarkers in Blood	Biomarkers in Saliva
Glial fibrillary acidic protein (GFAP)	Lactoferrin
Neurofilament light chain (NfL)	Melatonin
Phosphorylated tau (pTau) (in particular P-tau217)	Cortisol
Brain-derived growth factor (BDGF)	Cystatin B
Cytokines: IL-2, IL-5, IL-6, IL-8, IL-12, IL-13, IL-16, CCL3, CCL4, CCL17, CXCL10, TNFα, TNF-β, VEGF-C	
Aβ	

## Data Availability

No new data were created or analysed in this study. Data sharing is not applicable to this article.

## Abbreviations

The following abbreviations are used in this manuscript:

5mC	5-methylcytosine
5hmC	5-hydroxymethylcytosine
Aβ	amyloid-β peptide
ACE	angiotensin-converting enzyme
AD	Alzheimer’s disease
ALS	amyotrophic lateral sclerosis
APP	amyloid precursor protein
Bax	Bcl-2-associated X protein
Bcl-2	B-cell lymphoma 2
BDGF	brain-derived growth factor
BDNF	brain-derived neurotrophic factor
BPD	bipolar disorder
C9ORF72	chromosome 9 open reading frame 72
CAPNS1	calpain small subunit 1
CASK/Lin2	calcium/calmodulin-dependent serine protein kinase/liprin-2
CDK5	cyclin-dependent kinase 5
COX-2	cyclooxygenase-2
CRMP1	collapsin response mediator protein 1
CSNK2B	casein kinase 2β
CSF	cerebrospinal fluid
DISC1	disrupted in schizophrenia 1
DTNBP1	dystrobrevin binding protein 1
ECT	electroconvulsive therapy
FABP3	fatty acid binding proteins 3
FABP7	fatty acid binding proteins 7
FADD	FAS-associated death domain protein
FUS	fused in sarcoma
GEO	Gene Expression Omnibus
GFAP	glial fibrillary acidic protein
HPA	hypothalamic–pituitary–adrenal axis
HTT	huntingtin
MAM	methyl-azoxy-methanol
MAOA	monoamine oxidase A
MAPT	microtubule-associated protein tau
MDD	major depressive disorder
NfL	neurofilament light chain
NF-κB	nuclear factor κB
NGF	nerve growth factor
miRNA	micro-RNA
mTOR	mechanistic target of rapamycin
NMDA	N-methyl-D-aspartate
NO	nitrous oxide
NPAS3	neuronal PAS domain protein 3
NR3C1	nuclear receptor subfamily 3 group C member 1
Nrf2	nuclear factor erythroid 2-related factor 2
PARK2	Parkin (alternative name for *PRKN1*)
PD	Parkinson’s disease
PDZ	PSD95, Dlg1 and zo-1 (domain)
PDE3A	phosphodiesterase 3A
PINK1	PTEN-induced kinase 1
PPAR	peroxisome proliferator-activated receptor
PRKN	Parkin
PSEN1	presenilin 1
PTP4A2	protein tyrosine phosphatase 4A2
RARRES3	retinoic acid receptor responder 3
REST	repressor element 1 silencing transcription factor
SCZ	schizophrenia
SLC4A2	solute carrier family 4 member 2
SLC6A4	solute carrier family 6 member 4
SOD1	superoxide dismutase 1
TDP-43	TAR DNA-binding protein 43
TMS	transcranial magnetic stimulation
TNF-α	tumour necrosis factor α
TRIOBP	TRIO and F-actin binding protein
TrkA	tyrosine kinase receptor A
TrkB	tyrosine kinase receptor B
